# Transcriptome Analysis Reveals Genes Associated with Flooding Tolerance in Mulberry Plants

**DOI:** 10.3390/life13051087

**Published:** 2023-04-26

**Authors:** Jingtao Hu, Yanyan Duan, Junnian Yang, Liping Gan, Wenjing Chen, Jin Yang, Guosheng Xiao, Lingliang Guan, Jingsheng Chen

**Affiliations:** 1 College of Biology and Food Engineering, Chongqing Three Gorges University, Chongqing 404100, China; 20180016@sanxiau.edu.cn (J.H.);; 2 College of Teacher Education, Chongqing Three Gorges University, Chongqing 404100, China; 3 Tropical Crops Genetic Resources Institute, Chinese Academy of Tropical Agricultural Sciences, Haikou 571101, China

**Keywords:** mulberry, flooding stress, energy metabolism, ROS signaling, SSR

## Abstract

Mulberry (*Morus alba*), a widely distributed economic plant, can withstand long-term flooding stress. However, the regulatory gene network underlying this tolerance is unknown. In the present study, mulberry plants were subjected to submergence stress. Subsequently, mulberry leaves were collected to perform quantitative reverse-transcription PCR (qRT-PCR) and transcriptome analysis. Genes encoding ascorbate peroxidase and glutathione S-transferase were significantly upregulated after submergence stress, indicating that they could protect the mulberry plant from flood damage by mediating ROS homeostasis. Genes that regulate starch and sucrose metabolism; genes encoding pyruvate kinase, alcohol dehydrogenase, and pyruvate decarboxylase (enzymes involved in glycolysis and ethanol fermentation); and genes encoding malate dehydrogenase and ATPase (enzymes involved in the TCA cycle) were also obviously upregulated. Hence, these genes likely played a key role in mitigating energy shortage during flooding stress. In addition, genes associated with ethylene, cytokinin, abscisic acid, and MAPK signaling; genes involved in phenylpropanoid biosynthesis; and transcription factor genes also showed upregulation under flooding stress in mulberry plants. These results provide further insights into the adaptation mechanisms and genetics of submergence tolerance in mulberry plants and could aid in the molecular breeding of these plants.

## 1. Introduction

Many wild plant species and crops cannot withstand flooding. The presence of excess water seriously impairs the growth and development of terrestrial plants, disturbing the natural patterns of plant distribution. Based on the depth of water collection, flooding can be described as waterlogging or submergence. Flooding disrupts oxygen transport from the air into plant cells and thus produces a composite and complex form of stress in plants [1,2]. Plant species that are well adapted to flooding stress use either the escape strategy or the quiescence strategy to resist stress. The first strategy involves morphological alterations, such as aerenchyma formation in the roots and petiole elongation, which allow plants to absorb more oxygen. Meanwhile, in the second strategy, plants prolong underwater survival by conserving energy and launching tissue protection mechanisms [3].

The low-oxygen environments induced by submergence restrict oxygen uptake and cell respiration and thus lead to an energy shortage in plants. This energy shortage resulting from flooding stress can be managed by increasing starch degradation, sucrose catabolism, glycolysis, and ethanol fermentation [4]. The genes and enzymes associated with these pathways play pivotal roles in plant survival during submergence. Enzymes including amylases (AMY), sucrose synthase (SUS), and invertase (INV), which are involved in starch and sucrose catabolism, help plants in coping with oxygen deficiency [4,5,6]. Meanwhile, genes encoding phosphofructokinase (PFK), glyceraldehyde-3-phosphate dehydrogenase (GAPDH), pyruvate kinase (PK), alcohol dehydrogenase (ADH), fructose-bisphosphate aldolase (FBA), pyruvate decarboxylase (PDC), hexokinase (HXK), and enolase (ENO)—which are involved in the glycolysis and ethanol fermentation pathways—are always induced by flooding stress [7,8,9,10,11]. Additionally, biomolecules (lipids, proteins, RNA, and DNA) can be destroyed due to oxidative damage after exposure to adverse environmental conditions that lead to the production of excess reactive oxygen species (ROS) [12,13]. To alleviate this damage, plants recruit ROS-scavenging enzymes that mediate ROS homeostasis [14]. Furthermore, signaling pathways such as the phytohormone signaling, Ca^2+^, and mitogen-activated protein kinase (MAPK) signaling cascades are also triggered to reduce damage due to flooding stress [15].

Water dams are man-made structures built across waterways to control the flow of water. They are useful barriers and can improve navigation, control floods, and enable the generation of electric energy [16,17]. The Three Gorges Dam Reservoir (TGDR), which is located in the upper reaches of Chang Jiang in China, is one of the largest dams in the world. Since 2010, owing to floods and water storage, the water level of the TGDR has fluctuated between 145 and 175 m above sea level (ASL) annually [18]. Unfortunately, many native plants, which grow in water-level-fluctuation zones, have been unable to survive flood damage. This has caused environmental problems such as geological disasters, soil erosion, and biodiversity loss [19,20,21]. Restoring vegetation in the water-level-fluctuation zone of the TGDR, especially in the zone between 165 and 175 m ASL, is an effective and feasible strategy for mitigating these problems [22,23]. Woody plant species, such as *Morus alba* L., *Taxodium ascendens*, and *Salix variegata*, have been considered suitable candidates for the riverain forests because of their high resistance to flooding stress [23].

Mulberry (*Morus alba*) is a fast-growing plant. It can grow under a variety of conditions, and its leaves are widely used for sericulture [24]. Studies have shown that mulberry trees possess good adaptability against submergence stress. In the water-level-fluctuation zones of the TGDR, planted mulberries were found to survive submergence across several winters and showed good growth despite flooding stress. Some of the mulberry trees, which were planted 10 m below the water surface and were submerged for up to 200 days, could bloom in the spring after the water level dropped [23,25,26,27]. Some studies have investigated the physiological and molecular responses to submergence in *Arabidopsis*, rice, and some woody plants [28,29,30]. However, the genes that contribute to submergence tolerance in the mulberry plant remain unknown. To understand the mechanisms of short-term submergence tolerance in mulberry plants, we performed transcriptome analysis. Thus, we investigated the changes in their gene expression profiles under submergence stress in order to provide a basis for the breeding of flooding-tolerant varieties.

## 2. Materials and Methods

### 2.1. Plant Materials and Sample Collection

Mulberry variety Yu711 (*Morus alba*) plants were grown in the Ganning base of Chongqing Three Gorges Academy of Agricultural Sciences, Chongqing, China. Cuttings with an average diameter of 0.5 ± 0.1 cm were planted in pots (diameter, 30 cm; height, 23 cm), with each pot containing one plant. The plants were grown in a greenhouse (25 °C, 16 h day and 8 h night; 75% relative humidity). Two months later, the mulberry plants were subjected to flooding stress (FS). A water level of 10 cm above the soil surface was maintained during treatment. Two or three mature leaves were collected 0 d (Y711_0, FS0), 1 d (Y711_1, FS1), 3 d (Y711_3, FS3), and 7 d (Y711_7, FS7) after treatment. Then, all samples were immediately frozen in liquid nitrogen and stored at −80 °C until analysis. There were three independent biological replicates for each sampling point.

### 2.2. cDNA Library Construction and RNA Sequencing

The TRIzol reagent was used to isolate RNA from leaf samples according to the manufacturer’s instructions (Takara, Dalian, Liaoning, China). Agarose gel electrophoresis and the RNA Nano 6000 Assay Kit from the Bioanalyzer 2100 system (Agilent Technologies, Palo Alto, CA, USA) were then used to measure RNA integrity. Poly-T oligo-attached magnetic beads were used to separate mRNA from total RNA, and the mRNA was then fragmented using divalent cations. As soon as cDNA was synthesized, end-repaired, and adapter-ligated, library fragments were purified with a Beckman Coulter AMPure XP purification system (Beckman Coulter, Beverly, MA, USA). The Bioanalyzer 2100 system from Agilent was used to evaluate the quality of libraries. Finally, 150 bp paired-end reads were generated from each library via RNA sequencing (RNA-Seq) on the Illumina Novaseq 6000 platform.

### 2.3. Transcriptome Assembly and Data Analysis

To process raw data (raw reads) in the fastq format, we used in-house Perl scripts. We removed poly-N reads, adapters, and low-quality reads from the raw data to produce clean reads. The GC content, Q20, and Q30 of the clean data were calculated simultaneously. HISAT2 software was used to map clean reads to the mulberry genome (https://www.ncbi.nlm.nih.gov/genome/17692 (accessed on 24 June 2013)) [31]. We calculated the Fragments per Kilobase of transcript sequence per Million sequences (FPKM) value before analyzing gene expression, and FPKM ≥ 1 was set as the threshold for gene expression. To lower the false discovery rate, Benjamini–Hochberg adjustments were made to the obtained *p*-values. Genes were assessed using DESeq2, and differentially expressed genes (DEGs) were identified based on an adjusted *p*-value (*p*_adj_) ≤ 0.05 and |log2FoldChange| ≥ 1. DEG enrichment was analyzed based on Gene Ontology (GO) and Kyoto Encyclopedia of Genes and Genomes (KEGG) annotations using the Cluster Profiler R package. Heat maps were generated using TBtools [32].

### 2.4. Prediction of Simple Sequence Repeats

The microsatellite tool Krait v1.3.3 was used to identify expressed sequence tag–simple sequence repeat (EST-SSR) regions [33]. The minimum repeats for each perfect SSR type were set as follows: twelve for mononucleotides; seven for dinucleotides; five for trinucleotides; and four for tetra-, penta-, and hexa-nucleotides. SSR analysis was performed based on relative abundance and relative density, which indicate how many SSRs exist per Mb of the sequence analyzed and how long these SSRs are. The primers for all above-mentioned DEGs were designed based on the following criteria: primer length, 18–27 bp; product size, 100–300 bp; GC content, 30–80%; and annealing temperature, 58–65 °C.

### 2.5. Quantitative Reverse-Transcription PCR (qRT-PCR)

Using the RNAiso Plus reagent (Takara, China), total RNA was extracted from the leaves of three mulberry plants. M-MLV reverse transcriptase (Promega, Beijing, China) was used for the reverse transcription of RNA (1 g) into cDNA. Nine DEGs identified using transcriptome analysis were selected for qRT-PCR verification. qRT-PCR was performed on a Mastercycler^®^ ep realplex machine (Eppendorf, Hamburg, Germany). The reaction system included 0.5 μL primers, 1.0 μL cDNA, 5.0 μL 2 × SYBR Premix Go Taq (Promega, Beijing, China), and 3.5 μL DNase-free water. PCR parameters were as follows: 1 cycle of 5 min at 95 °C, followed by 40 cycles of denaturation for 15 s at 95 °C and annealing and elongation for 40 s at 60 °C. Melting curves were used to confirm specific qRT-PCR amplifications. The *Actin 3* gene of mulberry (GenBank accession number HQ163775.1) was used as the internal control gene [34]. Primer Premier 5 was used to design primers for qRT-PCR, and the sequences are provided in the Supplementary Materials (Table S1). qRT-PCR experiments for all samples were performed in triplicate.

### 2.6. Statistical Analysis

Data analysis was performed using Microsoft Excel 2019. To determine the significance of differences between mean values, one-way analysis of variance (ANOVA) with pairwise comparisons and Tukey’s honestly significant difference tests (*p* > 0.05) were conducted.

## 3. Results

### 3.1. RNA Sequencing and Assembly

In order to investigate the flooding tolerance mechanisms of mulberry plants, RNA-Seq analysis was performed. The Illumina Novaseq platform was used to construct and sequence 12 libraries, generating 540,843,510 raw reads. A total of 521,337,068 clean reads (96.4%) were obtained after filtering out low-quality sequences and adaptor contaminants. The clean read number per library ranged from 39.8 to 44.9 million, and the Q30 (base recognition accuracy, 99.9%) was greater than or equal to 90.43%. The mapping rates varied from 72.57% to 75.36% (Table 1).

Pearson correlation analysis demonstrated high reproducibility between biological replicates (R2 > 0.95) for all treatments (Figure S1A). Following principal component analysis (PCA) of leaf samples based on FPKM values, the four types of samples could be clearly separated, indicating a significant difference in gene expression profiles among these samples (Figure S1B).

### 3.2. Identification of DEGs under Flooding Stress

**Figure 1:**
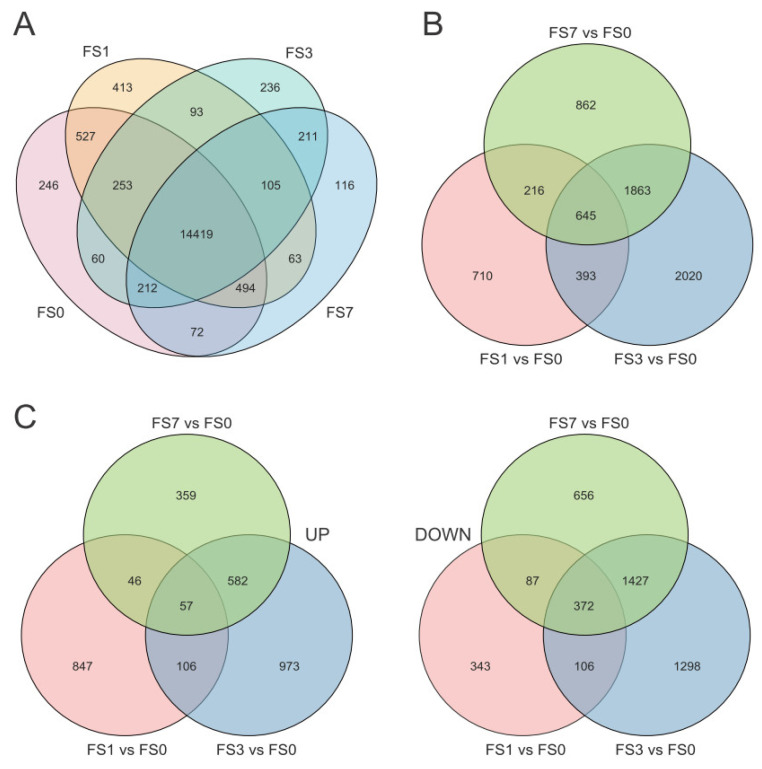
Overview of expressed genes and differentially expressed genes (DEGs) identified in mulberry leaves following flooding stress. (**A**) Venn diagram illustrating the number of genes expressed in control and submergence-treated mulberry leaves. (**B**) Venn diagram showing the overlap of all DEGs across the three comparison groups. (**C**) Venn diagram illustrating the overlap of up- and downregulated DEGs across the three comparison groups. FS0 indicates 0 d after submergence; FS1 indicates 1 d after submergence; FS3 indicates 3 d after submergence; and FS7 indicates 7 d after submergence.

Gene expression analysis was conducted based on transcriptome data. In total, 25,692 genes were found to be expressed. Of these, 16,283, 16,367, 15,589, and 15,692 gene transcripts were expressed in FS0, FS1, FS3, and FS7, respectively (Figure 1A).

Pairwise comparisons between stress (FS1, 3, 7) and control (FS0) plants were conducted to identify DEGs. In “FS1 vs. FS0”, a total of 1964 DEGs were identified (Figure 1B). Among these DEGs, 1056 were upregulated in response to one-day submergence, whereas 908 were downregulated (Figure 1C). In the “FS3 vs. FS0” group, 4921 DEGs (1718 upregulated, 3203 downregulated; Figure 1C) were identified (Figure 1B). After seven days of flooding stress (FS7 vs. FS0), a total of 3586 DEGs were identified (Figure 1B), of which 1044 and 2542 DEGs were up- and downregulated, respectively (Figure 1C).

**Figure 2:**
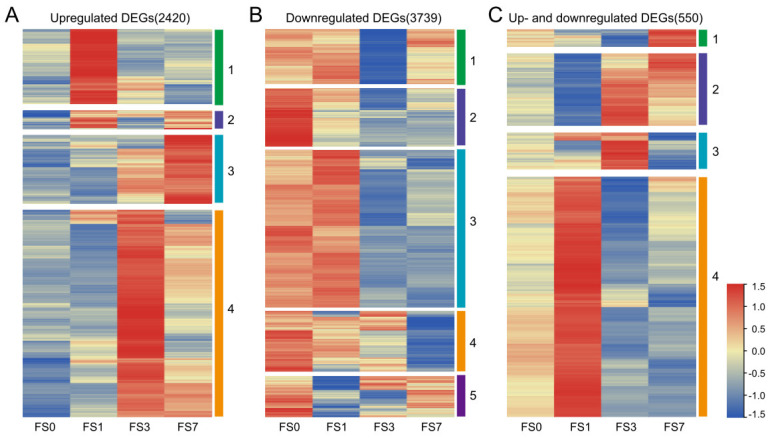
Expression heatmap of DEGs identified in mulberry plants after submergence stress. (**A**–**C**) Classification and expression patterns of 2420 upregulated DEGs (**A**), 3739 downregulated DEGs (**B**), and 550 up- and downregulated DEGs (**C**). DEGs were selected from all three comparison groups (FS1 vs. FS0, FS3 vs. FS0, FS7 vs. FS0).

A complete list of the DEGs identified in the three comparison groups is available in Tables S2 and S3. Among these DEGs, 2420 genes were upregulated after flooding stress, and 3739 genes were downregulated. Interestingly, 550 of the DEGs were upregulated at some stages of submergence stress and downregulated at others. Based on their expression levels, these DEGs were divided into different clusters (Figure 2).

### 3.3. GO Enrichment Analysis of DEGs

**Figure 3:**
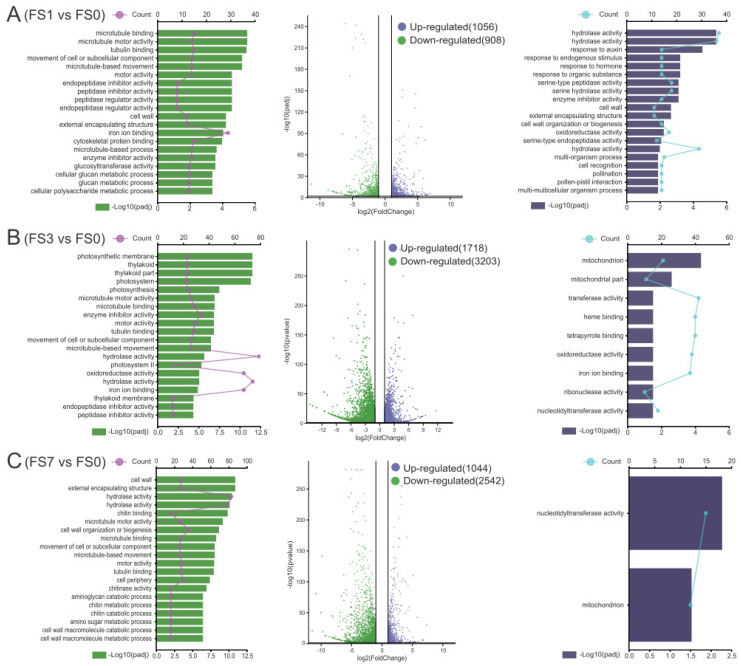
GO enrichment analysis of DEGs upregulated (**right**) and downregulated (**left**) in response to flooding stress at 1 d (**A**), 3 d (**B**) and 7 d (**C**). FS0, FS1, FS3, and FS7 indicate 0 d, 1 d, 3 d, and 7 d after submergence, respectively.

In order to explore the functions of DEGs under flooding conditions, GO enrichment analysis was performed. The DEGs in the three comparison groups—“FS1 vs. FS0”, “FS3 vs. FS0”, and “FS7 vs. FS0”—were significantly enriched in 63, 54, and 64 GO terms, respectively (Table S4). In “FS1 vs. FS0”, genes related to endogenous stimuli, hormones, oxidoreductase activity, cell wall organization, and other abiotic stressors were upregulated after submergence. In contrast, genes related to peptidase regulators, glucan metabolism, and microtubule motor activities were downregulated (Figure 3A). In the “FS3 vs. FS0” group, the upregulated genes were enriched in the mitochondrion, oxidoreductase activity, and heme binding and transferase activities. On the contrary, the downregulated genes were associated with photosynthesis, movement of cell or subcellular components, and hydrolase activities (Figure 3B). In the “FS7 vs. FS0” comparison group, upregulated genes were significantly enriched only in nucleotidyltransferase activity and in the mitochondrion, whereas downregulated genes were enriched in hydrolase activity, chitin metabolism, cell wall biogenesis, and cell wall macromolecule catabolism (Figure 3C).

### 3.4. KEGG Enrichment Analyses of DEGs

**Figure 4:**
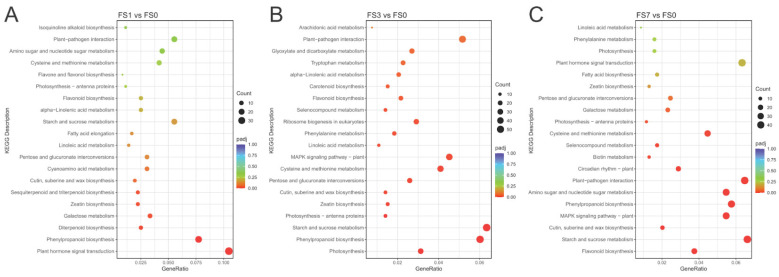
Top 20 enriched KEGG pathways for DEGs in the (**A**) “FS1 vs. FS0”, (**B**) “FS3 vs. FS0”, and (**C**) “FS7 vs. FS0” groups. The number of DEGs in each pathway is directly correlated to the size of the colored circles. The *p*_adj_ values shown in red are positive. FS0, FS1, FS3, and FS7 indicate 0 d, 1 d, 3 d, and 7 d after submergence, respectively.

DEGs identified at different stages of flooding stress were functionally annotated based on the KEGG database (Table S5). In the “FS1 vs. FS0” group, a total of 366 DEGs were successfully assigned to 100 KEGG pathways. The top five enriched pathways were as follows: transduction of hormone signals, biosynthesis of phenylpropanoid, diterpenoid biosynthesis, galactose metabolism, and zeatin biosynthesis (Figure 4A, Table S5). In the “FS3 vs. FS0” group, 937 DEGs were enriched in 111 KEGG pathways. The top five were photosynthesis, phenylpropanoid biosynthesis, metabolism of starch and sucrose, photosynthesis-antenna proteins, and zeatin biosynthesis (Figure 4B, Table S5). In the “FS7 vs. FS0” comparison, 701 DEGs were annotated to 111 KEGG pathways. Biosynthesis of flavonoids; metabolism of starch and sucrose; biosynthesis of cutin, suberin, and wax; biosynthesis of phenylpropanoid; and the MAPK signaling pathway were the top five enriched pathways (Figure 4C, Table S5). Among these pathways, those enriched for DEGs in at least two comparison groups were as follows: the biosynthesis of zeatin, biosynthesis of flavonoid, starch and sucrose metabolism, phenylpropanoid biosynthesis, and the MAPK signaling pathway.

### 3.5. DEGs Involved in ROS Signaling

**Figure 5:**
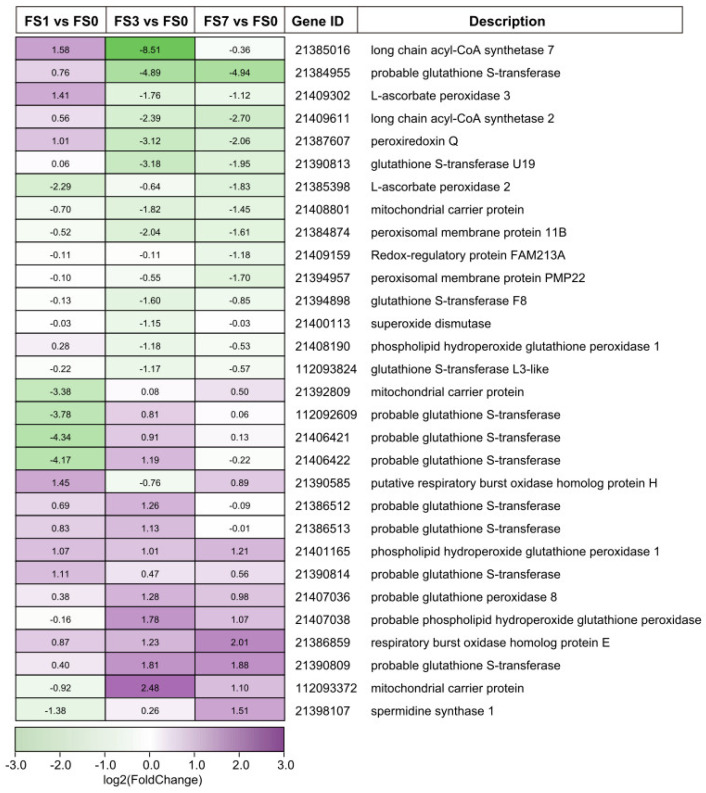
Expression profiles of genes related to ROS signaling at different stages of submergence. The numbers in the heatmap indicate the Log2(fold change). FS0, FS1, FS3, and FS7 indicate 0 d, 1 d, 3 d, and 7 d after submergence, respectively.

ROS play an important role in the response of plants to abiotic stresses. Several DEGs related to ROS signaling were identified in mulberry leaves at different stages of flooding stress. Of these, 15 DEGs were significantly upregulated after submergence treatment, and 8 were downregulated throughout flooding (Table S6). It is worth noting that two genes (21390585, 21386859) related to respiratory burst oxidase (RBOH), which promotes ROS production, showed elevated expression after submergence (Figure 5). Most scavenger enzyme-related genes, such as genes coding for glutathione peroxidase (GPX) (21407038, 21401165), ascorbate peroxidase (APX) (21409302), and glutathione S-transferase (GSTU), were also significantly upregulated after flooding stress. The results suggest that these genes play important roles in the flooding stress response of mulberry.

### 3.6. DEGs Involved in MAPK Signaling

The MAPK cascade plays a key role in regulating plant growth. In the KEGG enrichment analysis, 66 DEGs were annotated to the MAPK signaling pathway (Figure S2, Table S7). A total of 22 DEGs, such as genes encoding mitogen-activated protein kinase kinase 17 (MKKK17), MAP kinase 4 substrate 1 (MPK4), LRR receptor-like serine/threonine-protein kinase (FLS2), mulatexin (MLX), abscisic acid receptor (PYL4), and serine/threonine-protein kinases (SAPK2 and SAPK10), were remarkably upregulated during at least one of the submergence stages. The expression levels of 36 DEGs, including genes encoding epidermal patterning factor 1, pathogenesis-related protein 1 (PR), endochitinase 1, serine/threonine-protein kinase (OXI), and chitinase 9, were observably decreased following submergence stress. Genes related to abscisic acid receptor PYL4 and probable WRKY transcription factor 33 were first upregulated and then downregulated, while genes encoding abscisic acid receptor PYL11 and probable protein phosphatase 2C 8 showed the opposite trend.

### 3.7. DEGs Involved in Plant Hormone Signaling

**Figure 6:**
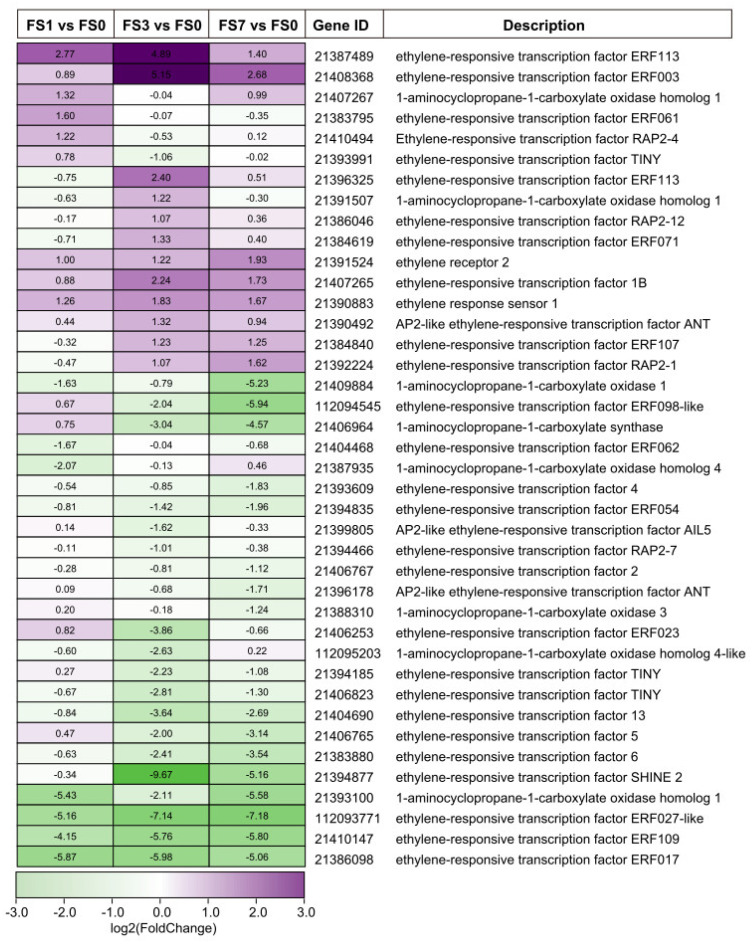
Heatmap of DEGs involved in ethylene signaling transduction. The numbers in the heatmap indicate the Log2(fold change). FS0, FS1, FS3, and FS7 indicate 0 d, 1 d, 3 d, and 7 d after submergence, respectively.

Hormones play a crucial role in controlling abiotic stress-induced signaling networks in plants. In this study, a total of 40 DEGs involved in ethylene signaling were detected on transcriptome analysis (Figure 6, Table S8). Among these DEGs, 12 genes coding for ethylene-responsive transcription factor (ERF), two genes coding for 1-aminocyclopropane-1-carboxylate oxidase (ACO), one gene coding for ethylene receptor (ETR), and one gene coding for ethylene response sensor (ERS) showed increased levels after flooding stress. Notably, three DEGs (21387489, 21391524, and 21390883) were significantly upregulated throughout submergence.

In addition, 11 genes related to zeatin biosynthesis also showed elevated expression after flooding stress (Figure S3A, Table S8). Nine DEGs related to auxins were identified in this study. Among these DEGs, one gene coding for auxin response factor, three encoding auxin-responsive proteins, and two related to auxin-induced protein were upregulated after flooding stress (Figure S3B, Table S8). Moreover, seven DEGs involved in abscisic acid (ABA) biosynthesis were identified in the analysis, more than half of which were upregulated (Figure S3C, Table S8).

### 3.8. DEGs Related to Energy Metabolism

In this study, the DEGs responsible for starch and sucrose metabolism, glycolysis and gluconeogenesis, metabolic cycling of citrate (TCA cycle), and oxidative phosphorylation were also examined. A total of 140 energy shortage response DEGs were detected during stress conditions (Figures S4–S6, Table S9). Among these DEGs, 76 were found to be involved in starch and sucrose metabolism; more than half of these genes were significantly activated by submergence. Among the upregulated genes, 34 showed markedly increased levels after submergence, including genes encoding beta-glucosidase, genes coding for alpha-/beta-amylase, and genes related to glucose-1-phosphate adenylyltransferase. Notably, DEGs encoding beta-glucosidase 24-like (112092781) and sucrose synthase (SUS) (21402491) were also obviously upregulated at all stages of flooding stress. The genes encoding sucrose synthase 6, hexokinase-3-like, beta-fructofuranosidase, and inactive beta-glucosidase as well as seven beta-glucosidase-encoding genes were first upregulated and then downregulated (Figure S4).

In total, 38 DEGs involved in the glycolysis/gluconeogenesis pathway were identified at different stages of flooding stress. Half of these were upregulated following submergence, including the genes coding for aldehyde dehydrogenase, putative glucose-6-phosphate 1-epimerase, aspartate aminotransferase, PDC, GAPDH, alcohol dehydrogenase-like 3, and PK (Figure S5).

Eighteen DEGs associated with the oxidative phosphorylation pathway were identified in this study. Three genes encoding cytochrome c oxidase assembly protein, one gene coding for V-type proton ATPase subunit e1, one gene encoding soluble inorganic pyrophosphatase, and one gene coding for protoheme IX farnesyltransferase were upregulated following submergence. It is worth noting that the gene coding for plasma membrane ATPase 4 (21399781) was markedly activated by flooding stress across different stages of flooding (Figure S6A). Only eight genes involved in the TCA cycle were differentially expressed after submergence. Four genes related to aconitate hydratase were downregulated at 1 d of submergence but were upregulated thereafter. Meanwhile, genes coding for ATP-citrate synthase beta chain protein (CS) and malate dehydrogenase (MDH) were inhibited by flooding stress at all stages of submergence (Figure S6B).

### 3.9. Other DEGs Detected in Mulberry Plants after Flooding Stress

Mitochondrial transcription termination factor (mTERF) proteins, which are encoded by nuclear genes, are involved in the regulation of gene transcription. In this study, 12 of 13 DEGs were significantly upregulated at 3 or/and 7 days of submergence stress (Figure S7A, Table S10).

Phenylpropanoids play an important role in plant adaptation to abiotic stresses by reducing ROS levels. In our study, 24 DEGs related to phenylpropanoid biosynthesis were identified. Among these DEGs, 11 were upregulated after submergence, including genes coding for cationic peroxidase 2, peroxidase 21, and beta-glucosidase. Meanwhile, 13 genes were downregulated in flooding-stressed leaves, such as genes coding for 4-coumarate-CoA ligase, phenylalanine ammonia-lyase, and cytochrome P450 98A2 (Figure S7B, Table S10).

Transcription factors (TFs) also play an important role in flooding stress. In our study, 273 differentially expressed transcription factor genes were identified, including 48 AP2-encoding genes, 13 bZIP-encoding genes, 15 GRAS-encoding genes, 24 bHLH-encoding genes, 16 homeobox-encoding genes, 11 HSF-encoding genes, 48 MYB-encoding genes, 23 NAC-encoding genes, and 25 WRKY-encoding genes (Table S10). Among them, 31, 64, and 41 genes were upregulated in FS1, FS3, and FS7, respectively (Table 2), indicating their important roles in the response of mulberry plants to flooding stress.

### 3.10. SSR Prediction

The sequences of all expressed genes identified on transcriptome analysis were analyzed for putative SSR markers using Krait software. A total of 12,501 perfect SSRs with an average length of 18.56 bp were identified, and the total length of perfect SSRs was 231,973 bp. The relative abundance and density were 323.09 loci and 5995.33 bp per Mb (Table S11), respectively. Mononucleotides (4351, 34.81%) were the most common type of repeat motifs, followed by dinucleotides (3898, 31.18%) and trinucleotides (3461, 27.69%). These three predominant motif types accounted for 93.68% of all motifs, whereas tetra-, penta-, and hexanucleotides represented only 6.32% of the total identified SSRs (Table S12). Of the identified motif sequences, the top five most frequent SSRs were A/T (34.56%), AG/CT (23.96%), AAG/CTT (10.72%), AT/AT (5.58%), and ATC/GAT (2.18%) (Table S13). SSRs with five tandem repeats were the most common in mulberry plants, followed by SSRs with seven and twelve tandem repeats (Table S14). A total of 409 primer pairs were generated from the 226 DEGs identified in this study (Table S15).

### 3.11. Validation of RNA-Seq Data by qRT-PCR

**Figure 7:**
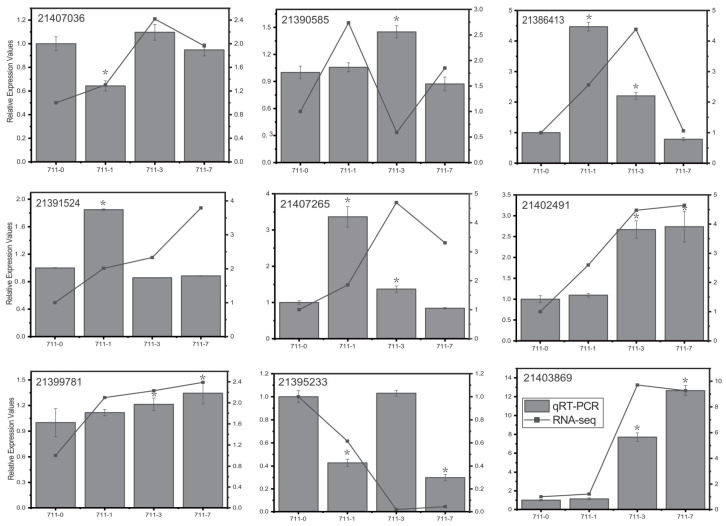
Validation and expression analysis of nine genes differentially expressed in mulberry leaves after submergence treatment. The actin gene (HQ163775.1) was used as the internal reference. The qRT-PCR results represent the mean ± standard deviation (SD) of three replicates. The RNA-seq results are shown as FPKM values. Asterisks (*) indicate a significant difference (*p* < 0.05) between stress (FS1, FS3, FS7) and control (FS0) plants.

The qRT-PCR results confirmed that the expression levels of genes encoding LRR receptor-like serine/threonine-protein kinase FLS2 (21386413), ethylene receptor 2 (21391524), ethylene-responsive transcription factor 1B (21407265), sucrose synthase (21402491), and starch phosphorylase (21403869) were observably increased following flooding stress (Figure 7). Meanwhile, a strong correlation between transcriptome sequencing data and qRT-PCR data was observed, validating our RNA-seq results.

## 4. Discussion

Flooding is a major environmental stress in natural and artificial ecosystems worldwide. Partial or complete submergence is detrimental to plant growth and development, and it thus influences food production and economic growth across the globe [35,36]. Several genes and related pathways have been implicated in the flooding stress response in plants [37,38]. In this study, DEGs induced in response to flooding stress in mulberry plants were detected using transcriptome analysis.

### 4.1. DEGs Associated with ROS Signaling

**Figure 8:**
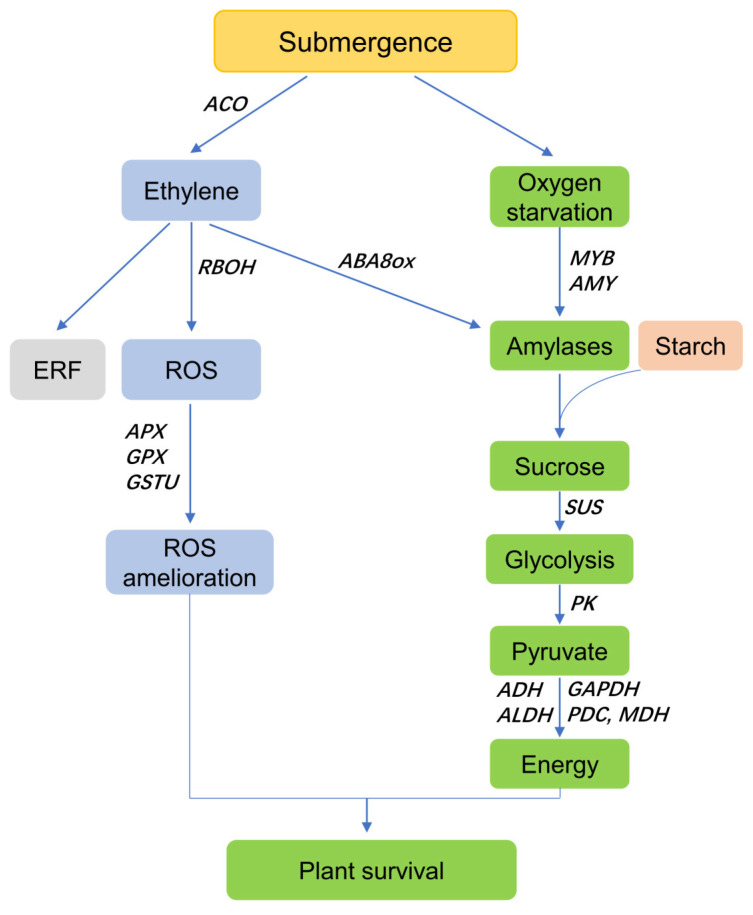
Proposed mechanism of partial submergence tolerance in mulberry.

Previous studies have demonstrated that flooding stress always leads to oxidative stress, rapidly triggering ROS-scavenging systems in plants [35,39]. ROS is also produced by RBOHs, which convert O_2_ to O_2_^−^. In plants, the expression of *RBOHs* is strongly upregulated under waterlogging stress [40,41]. In this study, two RBOH-encoding genes (21390585 and 21386859) were activated by flooding stress. The transcriptome analysis of bermudagrass following different degrees of submergence stress yielded similar findings [42]. In the presence of oxidative stress, many enzymes are recruited for ROS scavenging to maintain cellular homeostasis. Studies have shown that the overexpression of *DaAPX*, *GsGSTU*, and *MdSPDS1* can alleviate abiotic stress by enhancing ROS detoxification capacities in plants [43,44,45]. In the present study, the expression levels of an APX-encoding gene (21409302), three genes related to GSTU, and one gene coding for SPDS were significantly elevated after submergence. Interestingly, one study showed that the expression of the sAPX gene from *Myricaria laxiora* is significantly inhibited by flooding stress but is upregulated at the post-flooding recovery stage [46]. Together, our results indicate that genes related to ROS signaling may play an important role in the flooding stress response of mulberry (Figure 8).

### 4.2. DEGs Associated with Hormones

Phytohormones play critical roles in integrating multiple signaling pathways under abiotic stresses [47]. Recent studies have shown that ethylene, gibberellin, and auxin help plants adapt to flooding stress [48,49,50]. Ethylene biosynthetic enzymes, such as ACO and ACS, are activated by flooding stress. This leads to rapid ethylene accumulation, which promotes the formation of AR and aerenchyma in plants [40,51,52]. Ethylene-responsive transcription factors (ERFs), especially members of the ERF-VII family, play a vital role in resistance to waterlogging and submergence stresses in plants [53,54,55]. In this study, two genes (21407267 and 21391507) coding for 1-aminocyclopropane-1-carboxylate oxidase (ACO) and eleven DEGs encoding ERFs were upregulated after submergence. In addition, the transcription factor WRKY33, which plays a role in the response to submergence-induced hypoxia by upregulating *RAP2.2*, was also elevated in FS1 (Table S10). Interestingly, a study on *Myricaria laxiflora* found slightly different results. The genes coding for ERFs and ACO were significantly upregulated by flooding. However, the expression of *WRKY33* was downregulated [46]. In *Rumex palustris*, the accumulation of ethylene led to a massive reduction in ABA levels. Consistent with the reduction in ABA, the ABA-8-hydroxylase (an ABA breakdown enzyme)-encoding gene was activated [56]. In our study, two genes (21389777 and 21404773) related to ABA 8’-hydroxylase (ABA8ox) were upregulated after flooding stress (Figure S3C). Auxin has been implicated in the waterlogging stress response in several plant species [40,57,58]. Consistent with these findings, eight DEGs related to auxins were found to be upregulated in our study (Figure S3B). These results imply that DEGs associated with hormones are functionally involved in the submergence response in mulberry.

### 4.3. DEGs Associated with Energy Metabolism

Flooding and submergence stress directly limits oxygen exchange and thereby provokes an energy shortage in plants. A set of genes coding for enzymes involved in anaerobic metabolism are required for maintaining energy homeostasis and stress acclimation [42,59]. Flooding-activated AMY can promote the conversion of starch to glucose in plants [4]. In the current study, most DEGs annotated to the starch and sucrose metabolism pathway, including genes encoding beta-glucosidase, alpha-/beta-amylase, and glucose-1-phosphate adenylyltransferase, were significantly upregulated following submergence (Figure S4). Similar results have been reported in bermudagrass under different submergence stresses [42].

Previous studies indicate that genes coding for ADH and PDC play important roles in the ethanol fermentation pathway and contribute to waterlogging resistance in plants. The overexpression of *pdc1*, which encodes PDC, obviously enhances submergence tolerance in transgenic rice [60]. In addition, during waterlogging, *GmADH2*-transgenic soybean seeds germinate more readily [61]. Our results establish that genes related to ADH (21387053, 21384487, and 112094712) and PDC (21389594 and 21395264) are upregulated under flooding stress. Similarly, the expression levels of *PDCs* and *ADHs* are upregulated under waterlogging in cherry, cucumber, cotton, and soybean [62,63,64,65].

PDC can catalyze the conversion of pyruvate, the end-product of glycolysis, into acetyl-coA, which initiates the TCA cycle. The TCA cycle relies heavily on MDH, which converts malate into oxaloacetate [42]. In our study, the genes encoding MDH (21404632), aconitate hydratase (novel.1119, 21384011, 21411857, and 112094908), and ATPase (21399781 and 112090540) were upregulated. It is worth noting that several genes encoding ATP synthases (such as novel.547, 21399459, 21388005, 21408386, 112090832, and 21396694) were obviously downregulated, indicating weakened ATP synthesis and energy supply. Similar results were also observed in the transcriptome analysis of *Cynodon dactylon* [42].

### 4.4. Distribution of EST-SSRs in Mulberry

SSR markers have been used for diversity and DNA fingerprinting analysis owing to their stability and easy detection [66,67,68,69]. The main advantage of SSR markers obtained from transcriptome sequences is that the SSRs may be associated with functional genes and therefore directly linked to phenotypes [70]. Previous studies have identified many EST-SSRs from transcriptome sequencing data [71,72]. In the current study, a total of 12,501 perfect SSRs were identified from the transcriptome data. The number of SSRs obtained in this study was similar to that reported previously for *Morus laevigata* and *Morus serrata* [73,74]. In mononucleotide repeats, SSRs with the A/T motif were the most abundant, while in dinucleotide SSRs, the AG/CT sequence was predominant. These results were consistent with previous reports on *Morus alba* and *Lychnis kiusiana* [75,76]. The relative abundance and density of perfect SSRs in mulberry were 323.09 loci and 5995.33 bp per Mb, respectively, lower than the values reported for *Tinospora cordifolia* [77].

## 5. Conclusions

A comprehensive transcriptome profiling analysis was employed in the present study. KEGG enrichment analysis showed that the DEGs induced by flooding stress were primarily enriched in flavonoid biosynthesis, zeatin biosynthesis, starch and sucrose metabolism, the MAPK signaling pathway, and the biosynthesis of phenylpropanoid. Multiple genes involved in flooding stress in mulberry plants were identified. ROS-related genes, such as *RBOH*, *APX*, and *GSTU*, were significantly upregulated after submergence. More than 100 DEGs associated with energy shortage were also identified during the flooding stress response. The transcription levels of many genes, such as *AMY*, *SUS*, *PDC*, *ALDH*, *ADH*, and *MDH*, were significantly upregulated in mulberry plants following submergence. In addition, genes such as *ERF*, *ERS*, and *ABA8ox4* involved in hormone signaling and genes encoding AP2s, bZIPs, GRASs, NACs, homeobox, MYBs, bHLHs, and WRKYs were also upregulated in response to flooding stress. All these results suggest that the mulberry plant may utilize the escape strategy for survival following partial submergence (Figure 8). However, direct evidence for the function of these candidate genes in mulberry is still required to elucidate their exact roles during submergence stress. Overall, our results provide new insights into the flooding tolerance mechanisms of mulberry, and the candidate genes conferring flooding tolerance could be useful for the subsequent genetic improvement of mulberry and the development of submergence-resistant varieties.

## Figures and Tables

**Table 1:** Basic summary of RNA sequencing datasets and assembly statistics.

Sample	Raw Bases	Clean Reads	Q30 (%)	GC Content	Mapped Reads	Mapping Rate
Y711_0A	6.79G	43,491,174	90.90	46.73	32,773,537	75.36%
Y711_0B	6.90G	44,134,582	90.97	46.34	33,061,793	74.91%
Y711_0C	6.87G	43,715,556	90.76	46.35	32,528,709	74.41%
Y711_1A	6.80G	43,524,524	90.84	46.57	32,618,756	74.94%
Y711_1B	6.49G	41,667,112	90.43	46.85	31,207,395	74.90%
Y711_1C	6.80G	43,708,984	90.94	46.71	32,294,751	73.89%
Y711_3A	7.01G	44,921,118	91.10	45.97	32,755,910	72.92%
Y711_3B	6.18G	39,817,062	90.51	46.00	28,896,894	72.57%
Y711_3C	6.85G	44,029,322	90.81	45.91	32,117,560	72.95%
Y711_7A	6.89G	44,784,490	90.62	45.86	33,199,121	74.13%
Y711_7B	6.64G	43,138,614	91.05	45.82	31,792,726	73.70%
Y711_7C	6.90G	44,404,530	91.17	45.79	32,669,513	73.57%

Y711_0A, Y711_0B, and Y711_0C: mulberry leaves collected 0 d after flooding stress; Y711_1A, Y711_1B, and Y711_1C: mulberry leaves collected 1 d after flooding stress; Y711_3A, Y711_3B, and Y711_3C: mulberry leaves collected 3 d after flooding stress; Y711_7A, Y711_7B, and Y711_7C: mulberry leaves collected 7 d after flooding stress. Raw Bases: total number of bases in the raw data; Clean Reads: number of paired-end clean reads; Q30(%): percentage of bases with a Phred score greater than 30; GC Content: GC content of clean reads; Mapped Reads: number of mapped reads; Mapping Rate: proportion of mapped reads to clean reads.

**Table 2:** Differentially expressed transcription factor genes identified in the transcriptome analysis.

TF Family	FS1		FS3		FS7	
	Upregulated	Downregulated	Upregulated	Downregulated	Upregulated	Downregulated
AP2	2	14	12	23	6	25
bZIP	3	1	2	8	4	4
GRAS	0	4	4	8	2	9
bHLH	6	2	3	17	3	13
Homeobox	3	3	6	7	6	7
HSF	0	3	3	6	1	4
MYB	8	12	10	28	6	23
NAC	1	4	7	6	5	11
WRKY	3	8	6	15	1	18
MADS	3	1	4	1	1	2
YABBY	0	0	0	2	0	2
GATA	0	1	0	6	0	5
E2F	0	0	0	3	0	2
CO-like	0	1	2	4	1	4
NFYA	0	2	0	1	0	0
B3	0	3	0	5	0	5
Dof	0	0	2	2	3	1
C3H	1	1	1	3	0	1
TCP	1	0	1	1	1	1
ARF	0	0	1	0	1	0
ZF-HD	0	2	0	5	0	3
Total	31	62	64	151	41	140

## Data Availability

All the raw reads have been submitted to the NCBI SRA with the accession number PRJNA901038.

## Supplementary Materials

The following supporting information can be downloaded at: https://www.mdpi.com/article/10.3390/life13051087/s1. Figure S1. Pearson correlation coefficient analysis and principal component analysis (PCA) of RNA-seq samples. Figure S2. Heatmap of DEGs involved in MAPK signaling. Figure S3. Heatmap of DEGs involved in zeatin, auxin, and ABA signaling. Figure S4. Heatmap of DEGs involved in starch and sucrose metabolism pathways. Figure S5. Heatmap of DEGs related to glycolysis/gluconeogenesis. Figure S6. Expression profiles of DEGs related to oxidative phosphorylation and the TCA cycle. Figure S7. Expression profiles of DEGs related to mitochondrial function and phenylpropanoid biosynthesis. Table S1. Primer sequences used for qRT-PCR. Table S2. Complete list of downregulated DEGs identified in the three comparison groups. Table S3. Complete list of upregulated DEGs identified in the three comparison groups. Table S4. Gene ontology enrichment analysis of DEGs identified in plants under submergence stress. Table S5. Enriched KEGG pathways for DEGs in the comparison groups. Table S6. DEGs related to ROS signaling pathways. Table S7. DEGs related to MAPK signaling pathways. Table S8. DEGs related to hormone signaling pathways. Table S9. DEGs related to energy metabolism. Table S10. Other DEGs identified after flooding stress in mulberry. Table S11. Summary of detected sequences and perfect microsatellites. Table S12. Summary of perfect microsatellite types. Table S13. Abundance of each standard motif category. Table S14. Abundance of each repeat. Table S15. Primers generated for all the 226 DEGs.
