# Supplementary material for: Transcriptome Analysis Reveals Genes Associated with Flooding Tolerance in Mulberry Plants
Source: Life (Basel). 2023 Apr 26;13(5):1087. doi: 10.3390/life13051087 (PMC10222905; doi:10.3390/life13051087)
Supplement: Supplementary file 1 [file life-13-01087-s001.zip › life-2312327-supplementary/Supplementary material--Figure s1-s7.pdf]

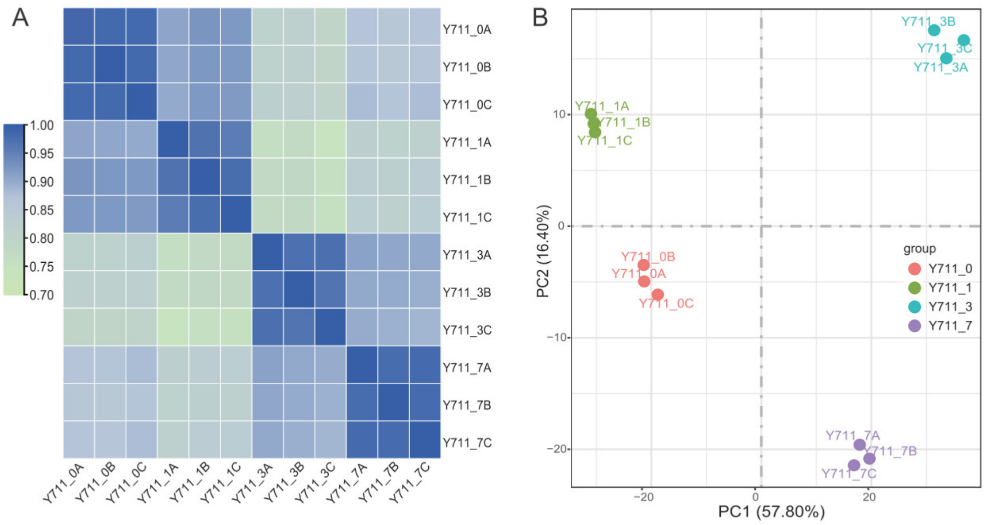

Figure S1. Pearson correlation coefficient and principal component analysis (PCA) of RNA-seq samples. (**A**) Pearson correlation coefficients of biological replicates. (**B)** PCA of individual biological replicates.

FS1 vs FS0	FS3 vs FS0	FS7 vs FS0	Gene ID	Description
	5.92	1.78	112091108	Mulatexin
0.44	4.97	0.85	112091105	mulatexin-like
1.35	2.14	0.09	21386413	LRR receptor-like serine/threonine-protein kinase FLS2
2.36	1.45	0.68	21402089	LRR receptor-like serine/threonine-protein kinase FLS2
0.77	0.50	1.32	21402449	REVERSION-TO-ETHYLENE SENSITIVITY1
1.26	1.83	1.67	21390883	ethylene response sensor 1
0.88	2.24	1.73	21407265	ethylene-responsive transcription factor 1B
0.59	1.82	2.17	21402711	serine/threonine-protein kinase SAPK2
1.00	1.22	1.93	21391524	ethylene receptor 2
0.87	1.23	2.01	21386859	respiratory burst oxidase homolog protein E
-0.93	2.71	2.17	21394326	mitogen-activated protein kinase kinase kinase 17
0.26	1.60	0.91	21385827	EIN3-binding F-box protein 1
0.11	1.49	-0.10	21395281	Abscisic acid receptor PYL4
-0.39	1.04	0.20	21385232	nucleoside diphosphate kinase 1
-0.19	1.01	0.58	21396411	serine/threonine-protein kinase SAPK10
3.86	-0.77	0.19	112092684	basic form of pathogenesis-related protein 1-like
3.75	0.54	0.50	21385416	Protein MAP kinase 4 substrate 1
-0.98	0.49	-2.33	112095318	Acidic endochitinase Q
-0.58	0.41	-3.99	112091100	mulatexin-like
0.20	1.71	-2.65	21390361	Abscisic acid receptor PYL4
0.42	2.07	-0.90	21411796	acidic endochitinase Q
-2.24	-1.66	1.52	21387335	abscisic acid receptor PYL11
-0.24	-1.02	-0.37	21401574	LRR receptor-like serine/threonine-protein kinase ERECTA
-0.48	-1.01	-0.55	21402281	mitogen-activated protein kinase kinase 9
-0.36	-0.90	-1.51	21409949	mitogen-activated protein kinase kinase 2
-0.30	-0.67	-1.29	21390739	mitogen-activated protein kinase kinase kinase NPK1
-0.37	-1.07	-1.04	21407041	Probable copper-transporting ATPase HMA5
0.04	-1.02	-1.21	21393504	disease resistance protein SUMM2
-0.07	-0.91	-1.01	21384958	mitogen-activated protein kinase kinase 5
0.59	-1.48	-1.76	21411794	Endochitinase
0.52	-1.72	-1.78	21407042	probable copper-transporting ATPase HMA5
0.41	-0.28	-2.16	21391453	mitogen-activated protein kinase kinase kinase 18
0.19	-0.53	-2.14	112091117	mulatexin-like
-0.86	-0.83	-1.74	21411569	Endochitinase 1
-0.58	-0.46	-1.90	21394469	mitogen-activated protein kinase 3
-0.20	-0.83	-2.15	21397505	Endochitinase 1
-0.23	-0.88	-2.47	21396732	pathogenesis-related protein 1
0.03	-1.35	-2.11	112091103	Mulatexin
0.16	-0.98	-1.86	21412808	mulatexin
0.61	-2.79	-0.98	21406208	transcription factor MYC2
-0.25	-2.32	-0.39	21386266	probable protein phosphatase 2C 51
-0.55	-2.15	-1.40	21409375	LRR receptor-like serine/threonine-protein kinase FLS2
-0.62	-2.18	-0.95	21384903	probable protein phosphatase 2C 51
0.70	-1.14	-0.05	21397332	mitogen-activated protein kinase kinase kinase 18
0.48	-1.02	-0.09	21394458	mitogen-activated protein kinase homolog NTF6
0.28	-1.16	-0.04	21406151	serine/threonine-protein kinase SAPK3
-0.35	-1.58	0.25	21408017	probable protein phosphatase 2C 24
-0.28	-1.35	1.04	21387410	probable protein phosphatase 2C 8
-5.03	-10.89	-9.35	112095323	Endochitinase 3
0.77	-6.25	-0.74	21395166	mitogen-activated protein kinase kinase kinase 18
-0.39	-4.69	-2.39	21390989	mitogen-activated protein kinase kinase kinase 18
0.04	-3.53	-1.56	21394000	mitogen-activated protein kinase kinase kinase 1
-0.31	-3.80	-1.92	21396136	probable protein phosphatase 2C 75
0.75	-3.04	-4.57	21406964	1-aminocyclopropane-1-carboxylate synthase
1.13	-2.44	-3.65	21397160	probable WRKY transcription factor 33
-0.05	-2.85	-3.07	21411601	mulatexin
-0.01	-2.83	-3.47	21387251	pathogenesis-related protein PRB1-3
-0.16	-2.19	-2.91	21394408	probable WRKY transcription factor 26
-0.59	-2.64	-2.94	21398911	serine/threonine-protein kinase OXI1
-1.49	-2.09	-2.80	21411792	Chitinase 9
-0.64	-1.46	-2.51	21397503	Endochitinase 1
-0.92	-1.31	-3.22	112090313	mitogen-activated protein kinase kinase kinase YODA
-1.48	-5.22	-5.26	21408360	Protein EPIDERMAL PATTERNING FACTOR 1
-1.92	-5.70	-5.75	21387023	EPIDERMAL PATTERNING FACTOR-like protein 6
-4.48	-2.17	-4.64	112095316	Endochitinase 1
-3.68	-2.04	-3.70	21387250	pathogenesis-related protein 1

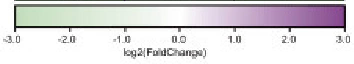

Figure S2. Heatmap of DEGs involved in MAPK signaling. The numbers in the heatmap indicate the Log2(fold change). FS0, FS1, FS3, and FS7 indicate 0 d, 1 d, 3 d, and 7 d after submergence, respectively.

	FS1 vs FS0	FS3 vs FS0	FS7 vs FS0	Gene ID	Description
A	0.03	2.26	2.37	21394046	7-deoxyloganetin glucosyltransferase
	1.69	1.29	0.20	21391993	cytokinin dehydrogenase 6
	2.20	2.83	0.69	21405415	UDP-glycosyltransferase 73C6
	-0.93	2.33	-2.09	21400185	7-deoxyloganetin glucosyltransferase
	-0.30	1.93	-0.76	21405641	scopoletin glucosyltransferase
	0.04	1.39	-1.39	21395926	7-deoxyloganetin glucosyltransferase
	-0.25	1.07	-1.69	21404740	cytokinin dehydrogenase 5
	-0.81	1.11	1.12	21401563	cytokinin dehydrogenase 7
	-1.31	1.53	0.45	21394045	7-deoxyloganetin glucosyltransferase
	-1.22	1.23	-0.11	21399598	zeatin O-xylosyltransferase
	-0.87	1.26	0.26	21405414	UDP-glycosyltransferase 73C1
	-1.42	0.98	-1.04	21407760	adenylate isopentenyltransferase 5
	-1.45	0.05	0.06	21393340	adenylate isopentenyltransferase 5
	-1.10	0.37	0.04	21399600	zeatin O-xylosyltransferase
	0.49	-2.14	-0.01	21399606	cytokinin dehydrogenase 1
	-3.21	-2.21	-2.67	21409909	cytokinin dehydrogenase 3
	0.06	-3.73	-4.74	21395924	7-deoxyloganetin glucosyltransferase
	-0.04	-0.93	-3.89	21405158	linamarin synthase 2
	-0.71	-1.22	-3.80	21405160	linamarin synthase 1
B	1.33	1.36	0.75	21407314	auxin-responsive protein IAA4
	-0.45	2.25	1.08	21388604	auxin response factor 5
	1.55	-2.33	-1.32	21395766	auxin-induced protein IAA4
	0.66	-1.55	-0.91	21403062	auxin transporter-like protein 3
	1.13	-1.39	-1.09	21398572	auxin-induced protein X10A
	1.12	-1.85	-1.08	21407698	auxin transporter-like protein 2
	1.12	-3.27	-2.52	21395456	auxin-responsive protein SAUR21
	1.98	-2.80	-3.44	21384057	auxin-induced protein 15A
	1.80	-3.06	-3.93	21395460	auxin-responsive protein SAUR50
C	-0.11	-7.20	-5.66	21407981	abscisic acid 8'-hydroxylase 2
	0.90	2.47	1.94	21404773	abscisic acid 8'-hydroxylase 4
	-0.52	1.81	0.62	21389777	abscisic acid 8'-hydroxylase 4
	-0.06	0.27	1.10	21404497	9-cis-epoxycarotenoid dioxygenase NCED3
	1.39	-1.88	0.30	21407351	9-cis-epoxycarotenoid dioxygenase NCED1
	-2.49	-1.18	-1.14	21392134	abscisic acid 8'-hydroxylase 1
	-1.32	-2.79	-1.66	21410144	abscisic acid 8'-hydroxylase 4

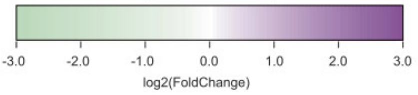

Figure S3. Heatmap of DEGs involved in zeatin (A), auxin (B), and ABA (C) signaling. The numbers in the heatmap indicate the Log2(fold change). FS0, FS1, FS3, and FS7 indicate 0 d, 1 d, 3 d, and 7 d after submergence, respectively.

FS1 vs FS0	FS3 vs FS0	FS7 vs FS0	Gene ID	Description
1.97	2.48	4.78	112092781	beta-glucosidase 24-like
0.41	3.65	3.98	21401539	glucose-1-phosphate adenylyltransferase large subunit 1
0.27	3.28	3.22	21403869	starch phosphorylase L
0.92	3.28	3.14	21404904	alpha-glucan phosphorylase
-0.79	3.86	2.65	21394903	probable alpha-amylase 2
0.74	3.22	1.85	21408604	alpha-glucosidase
-0.04	2.65	-0.12	21400109	glutamate dehydrogenase 2
-0.52	2.15	0.30	21389771	alpha-amylase
0.04	2.11	0.25	21392687	beta-glucosidase BoGH3B
-1.23	0.85	-0.20	21403957	alpha-amylase 3
-0.63	1.11	0.80	21406385	fructokinase-like 1
-0.75	1.32	0.26	21390946	beta-glucosidase 3
-1.08	1.41	0.34	112094672	putative beta-glucosidase 6
1.27	0.66	1.58	21401263	beta-amylase
1.62	0.92	1.82	novel.355	beta-amylase
0.78	1.59	3.00	21402108	Beta-fructofuranosidase, insoluble isoenzyme 1
1.37	2.16	2.22	21402491	sucrose synthase
0.85	1.90	1.98	21404777	glucose-1-phosphate adenylyltransferase large subunit 1
-0.80	1.59	1.72	21395116	soluble starch synthase 1
-0.87	1.84	2.01	21407567	1,4-alpha-glucan-branching enzyme 1
-0.20	1.51	2.26	21396955	starch branching enzyme I
-0.29	2.18	2.23	21395691	1,4-alpha-glucan-branching enzyme 3
0.02	2.20	2.15	21407750	isoamylase 2
0.59	1.19	0.80	21400758	trehalose-6-phosphate synthase 5
0.58	1.10	0.57	21409854	probable fructokinase-6
0.45	1.13	0.51	21410504	4-alpha-glucanotransferase DPE2
-0.05	1.64	0.44	21402976	amylomaltase
0.19	1.44	0.22	21404893	beta-glucosidase BoGH3B
-0.32	1.77	1.38	21398473	granule-bound starch synthase 1
-0.20	1.20	0.98	21394396	sucrose synthase 2
-0.05	1.47	1.16	21391606	isoamylase 1
-0.09	1.27	1.24	21386355	probable fructokinase-7
0.05	1.22	1.26	21409103	fructokinase-like 2
-0.58	0.43	1.28	21396372	isoamylase 3
0.23	0.58	1.46	21390193	trehalose-6-phosphate synthase 6
0.27	1.01	1.12	21390301	glucose-1-phosphate adenylyltransferase small subunit 2
-0.71	-5.73	-4.50	21395233	endoglucanase 8
-0.05	-6.20	-0.41	21401980	acid beta-fructofuranosidase
2.09	-6.32	-2.51	21412606	beta-glucosidase 17
4.49	-1.06	1.41	112090497	beta-glucosidase 17-like
-1.47	-0.24	-1.08	21384482	glucan endo-1,3-beta-glucosidase 3
-0.89	-0.92	-2.85	21406136	beta-amylase 3
-0.17	-1.55	-1.98	21403872	UDP-glucose 6-dehydrogenase 3
-0.77	-1.57	-1.69	21385703	trehalose-6-phosphate synthase 9
-1.20	-1.44	-1.95	21409810	glucan endo-1,3-beta-glucosidase 2
0.91	1.40	-1.15	21400400	beta-glucosidase 44
1.24	0.29	-0.40	112091302	beta-glucosidase 12-like
1.32	-0.25	-0.29	112095313	hexokinase-3-like
-0.21	-0.36	-1.65	21403045	nitrate reductase
0.46	-0.71	-1.50	21404217	trehalose-6-phosphate synthase 7
0.13	-0.54	-1.15	21390077	UDP-glucose 6-dehydrogenase 1
0.16	-1.11	-0.01	21386357	phosphoglucomutase
0.63	-1.05	-0.25	21402429	beta-fructofuranosidase, insoluble isoenzyme CWINV1
-0.53	-1.18	-0.24	21389873	probable sucrose-phosphatase 2
-0.20	-1.17	-0.73	21386919	acid beta-fructofuranosidase 1
-0.27	-1.07	-0.90	21395194	trehalose-6-phosphate synthase 11
0.07	-1.03	-1.14	21398701	trehalose-6-phosphate synthase 1
0.25	-1.25	-0.90	112091393	beta-glucosidase 12-like
1.51	-1.58	-0.55	21407811	sucrose synthase 6
1.17	-1.18	-0.47	21408363	probable inactive beta-glucosidase 14
1.07	-1.04	-1.14	112090789	beta-glucosidase 18-like
0.79	-1.54	-1.09	112094920	putative beta-glucosidase 17
1.19	-0.35	-2.06	21388593	beta-fructofuranosidase
1.84	-1.26	-1.80	21397857	beta-glucosidase BoGH3B
3.03	-3.47	-0.45	novel.653	beta-glucosidase 16
1.40	-4.11	-2.20	21385981	beta-glucosidase 17
0.74	-3.80	-1.34	21389547	UDP-glucose 6-dehydrogenase 1
0.94	-2.52	-3.46	21404409	probable trehalose-phosphate phosphatase D
0.35	-3.42	-2.62	21386920	trehalose-phosphate phosphatase A
0.95	-3.30	-2.61	21387408	endoglucanase 6
0.75	-2.68	-0.71	112091396	beta-glucosidase 12-like
0.92	-2.62	-1.73	21401505	endoglucanase 24
0.17	-2.69	-1.83	21401982	probable trehalose-phosphate phosphatase F
-0.41	-3.96	-1.34	21402017	beta-glucosidase 11
-0.02	-2.15	-1.05	112091395	beta-glucosidase 12-like
-0.79	-2.19	-0.90	112091869	probable trehalose-phosphate phosphatase C

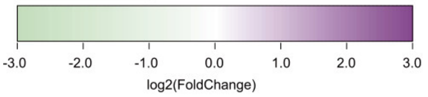

Figure S4. Heatmap of DEGs involved in starch and sucrose metabolism pathways. The numbers in the heatmap indicate the Log2(fold change). FS0, FS1, FS3, and FS7 indicate 0 d, 1 d, 3 d, and 7 d after submergence, respectively.

FS1 vs FS0	FS3 vs FS0	FS7 vs FS0	Gene ID	Description
-0.29	-8.40	-3.21	21398387	aldehyde dehydrogenase
0.08	-6.89	-4.50	21384564	pyruvate decarboxylase 2
-5.69	0.11	-3.40	112091578	alcohol dehydrogenase-like 1
-1.26	2.55	0.91	21399168	phosphoenolpyruvate carboxykinase (ATP)
-1.05	1.70	1.55	21396538	aldehyde dehydrogenase
-0.83	2.00	1.38	21403466	putative glucose-6-phosphate 1-epimerase
-0.35	1.29	1.21	21398109	aldehyde dehydrogenase
-0.17	1.71	1.35	21385564	enolase
-0.02	1.14	0.62	112092871	pyruvate kinase 1
0.14	1.44	1.06	21403699	aldehyde dehydrogenase
-0.01	1.65	0.75	21389594	pyruvate decarboxylase 1
-0.26	2.12	0.39	21385919	pyruvate kinase
-0.33	1.98	1.06	21390673	aspartate aminotransferase
-0.48	1.84	0.73	21396462	aspartate aminotransferase 3
-2.00	0.08	-0.61	21403701	aldehyde dehydrogenase
-0.55	1.54	0.28	112094712	alcohol dehydrogenase 3-like
-0.17	1.16	0.03	21387053	alcohol dehydrogenase 1
-0.29	1.20	0.04	21384487	alcohol dehydrogenase 1
0.30	1.03	-0.33	21408222	phosphoenolpyruvate carboxykinase (ATP)
0.17	1.11	-0.08	21395264	pyruvate decarboxylase 1
-0.23	1.24	-0.47	21406686	glyceraldehyde-3-phosphate dehydrogenase
0.08	1.09	-0.67	21390149	glyceraldehyde-3-phosphate dehydrogenase
1.39	0.30	-0.63	21398466	alcohol dehydrogenase-like 3
1.24	-0.86	-1.09	112094039	aldehyde dehydrogenase
1.30	-1.08	-0.46	21388698	pyruvate kinase 1
-0.58	-0.77	-2.04	21386896	pyruvate kinase isozyme A
-0.21	-0.62	-2.54	21396969	fructose-bisphosphate aldolase 6
0.04	-0.18	-2.89	21396966	fructose-bisphosphate aldolase 6
-1.13	-1.84	-1.09	21394558	pyruvate, phosphate dikinase
0.61	-1.07	-0.40	21386607	putative glucose-6-phosphate 1-epimerase
0.01	-1.27	-0.23	21388958	ATP-dependent 6-phosphofructokinase 3
0.11	-1.18	-0.73	21399424	pyruvate dehydrogenase complex component E2 4
-0.08	-1.08	-0.54	21406394	fructose-1
0.23	-1.74	-1.66	21406393	fructose-1
0.45	-1.44	-1.11	21407558	glyceraldehyde-3-phosphate dehydrogenase B
0.36	-1.43	-0.92	21404496	pyruvate dehydrogenase E1 component subunit beta-3
0.19	-1.89	-0.84	21400859	fructose-1
-0.11	-1.41	-0.90	novel.1190	plastidial pyruvate kinase 2
-0.23	-1.70	-1.12	21403127	fructose-bisphosphate aldolase
-0.29	-1.71	-1.03	21402005	L-lactate dehydrogenase A

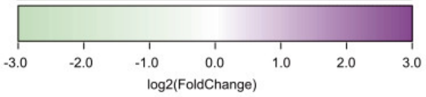

Figure S5. Heatmap of DEGs related to glycolysis/gluconeogenesis. The numbers in the heatmap indicate the Log2(fold change). FS0, FS1, FS3, and FS7 indicate 0 d, 1 d, 3 d, and 7 d after submergence, respectively.

	FS1 vs FS0	FS3 vs FS0	FS7 vs FS0	Gene ID	Description
A	-0.03	-2.53	0.52	21410359	cytochrome b-c1 complex subunit 8
	0.54	-1.25	-0.42	novel.547	ATP synthase subunit alpha
	0.05	-1.52	-0.94	21399459	ATP synthase delta chain
	0.07	-1.37	-0.79	21388005	ATP synthase subunit b
	-0.51	-1.15	-0.70	21408386	ATPase 9
	-0.18	-1.41	-1.05	21396694	ATP synthase gamma chain
	-0.30	-1.20	-1.21	21408817	leghemoglobin reductase
	-1.13	0.75	-0.39	112090832	V-type proton ATPase subunit F
	-1.17	0.66	0.53	21385126	plasma membrane ATPase 2
	-1.71	0.49	0.62	novel.400	plasma membrane ATPase 1
	-1.51	0.64	0.51	112091054	plasma membrane ATPase 1-like
	1.00	1.92	-0.18	112090540	V-type proton ATPase subunit e1
	0.07	1.10	-0.36	21392266	soluble inorganic pyrophosphatase 4
	-0.27	1.49	0.34	21408913	cytochrome c oxidase assembly protein cox19
	-0.12	2.01	2.02	21384539	cytochrome c xidase assembly protein cox11-2
	1.06	1.16	1.26	21399781	plasma membrane ATPase 4
	0.30	1.50	0.86	21406515	cytochrome c oxidase assembly protein COX15
	0.62	1.13	0.78	21390710	protoheme IX farnesyltransferase
B	-3.38	2.77	1.86	novel.1119	aconitate hydratase
	-2.15	1.76	0.86	21384011	aconitate hydratase
	-2.35	2.17	0.95	21411857	aconitate hydratase
	-2.33	2.28	0.64	112094908	aconitate hydratase
	-0.40	-1.27	-4.21	21392590	ATP-citrate synthase beta chain protein 2
	-0.20	-1.77	-1.07	21384723	malate dehydrogenase
	1.55	-1.31	-0.60	21404632	malate dehydrogenase

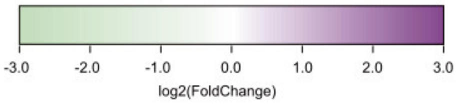

Figure S6. Expression profiles of DEGs related to oxidative phosphorylation (A) and the TCA cycle (B). The numbers in the heatmap indicate the Log2(fold change). FS0, FS1, FS3, and FS7 indicate 0 d, 1 d, 3 d, and 7 d after submergence, respectively.

	FS1 vs FS0	FS3 vs FS0	FS7 vs FS0	Gene ID	Description
A	0.56	-2.70	-1.74	21394692	transcription termination factor MTEF1
	-7.94	3.30	1.77	21404681	transcription termination factor MTEF18
	-1.80	4.50	3.16	21404680	transcription termination factor MTEF18
	0.32	2.57	2.46	21397005	transcription termination factor MTERF2
	0.60	1.68	2.19	21403386	uncharacterized mTERF
	-0.65	0.64	1.27	21400626	transcription termination factor MTERF5
	-0.59	-0.06	1.20	21392830	transcription termination factor MTERF9
	0.40	1.91	0.98	112093229	uncharacterized mTERF
	-0.82	1.09	0.64	21386792	transcription termination factor MTERF8
	-0.48	1.14	0.80	112091973	transcription termination factor MTERF15%
	-0.43	1.46	0.67	112091964	transcription termination factor MTERF15
	-0.13	0.85	1.49	21401514	transcription termination factor MTERF2
	0.25	1.10	1.13	21401009	transcription termination factor MTEF18
B	2.09	-6.32	-2.51	21412606	beta-glucosidase 17
	4.07	2.08	1.42	21400231	cationic peroxidase 2
	0.84	0.49	1.23	112094400	beta-glucosidase 11
	1.33	1.09	0.63	21407160	peroxidase 21
	0.91	1.40	-1.15	21400400	beta-glucosidase 44
	1.21	0.79	-3.33	21406195	caffeic acid 3-O-methyltransferase
	-0.32	-1.89	-1.88	21402620	4-coumarate--CoA ligase 2
	-0.46	-2.13	-2.03	21407115	phenylalanine ammonia-lyase 1
	-0.40	-1.88	-2.18	21407113	phenylalanine ammonia-lyase 1
	-0.99	-1.54	-2.11	21410772	feruloyl CoA ortho-hydroxylase 1
	-0.62	-1.60	-1.92	112091386	feruloyl CoA ortho-hydroxylase 1-like
	-1.28	-1.17	-1.74	21402709	peroxidase 17
	-0.27	-1.11	-0.97	21410476	cytochrome P450 98A2
	-1.07	-1.40	-0.53	112090580	feruloyl CoA ortho-hydroxylase 1-like
	1.40	-4.11	-2.20	21385981	beta-glucosidase 17
	1.21	-2.94	-2.52	21411521	berberine bridge enzyme-like 15
	0.63	-2.92	-3.40	21412376	feruloyl CoA ortho-hydroxylase 1
	0.53	-3.28	-3.09	112091846	feruloyl CoA ortho-hydroxylase 1-like
	0.55	-2.20	-0.70	21396240	phenylalanine ammonia-lyase
	0.52	-1.23	-0.75	21399565	probable cinnamyl alcohol dehydrogenase 9
	0.60	-1.59	-0.48	21401056	shikimate O-hydroxycinnamoyltransferase
	1.24	-0.86	-1.09	112094039	aldehyde dehydrogenase family 2 member C4-like
	1.84	-1.26	-1.80	21397857	beta-glucosidase BoGH3B
	2.27	-1.19	-1.28	21409963	phenylalanine ammonia-lyase

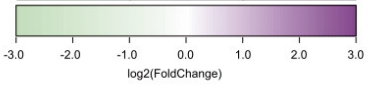

Figure S7. Expression profiles of DEGs related to mitochondrial function (A) and phenylpropanoid biosynthesis (B). The numbers in the heatmap indicate the Log2(fold change). FS0, FS1, FS3, and FS7 indicate 0 d, 1 d, 3 d, and 7 d after submergence, respectively.
